# Thoracic Aneurism

**Published:** 1887-02

**Authors:** H. P. Wenzel

**Affiliations:** Ex-President of the Rock River Medical Society; 296 West Water Street, Milwaukee


### THORACIC ANEURISM, WITH A CASE OF DISSECTING ANEURISM OF THE INFERIOR SURFACE OF THE AORTIC ARCH.

*By* H. P. WENZEL, M.D., *Ex-President of the Rock River Medical Society.*

[Read before the Rock River Medical Society on November 9th, 1886. Published by request of the Society.]

It is said we learn more by "mistakes and failures than by success;" therefore I present for your consideration Thoracic Aneurism, with a case under my care which proves that there is "no absolute sign in aortic aneurism." My diagnosis was not verified on autopsy. The specimen could not be obtained.

On July 26th, 1886, L. Y., an actress, a native of Virginia, consulted me. She has been on the stage sixteen years, married sixteen years, is 32 years old, 5 feet high, and weighs 135 pounds. She was never pregnant, and her only sickness was a bad cold ten years ago. She suffers from obstinate constipation, palpitation, sometimes a severe pain under the left breast, which frequently shifts to the right temple. Her sleep is broken, and she is restless; tires easily, and on exertion gets "winded." Her appetite is good; her menses are regular, and her urine normal. She sweats easily.

*Status præsens:* She is well developed, has gray eyes, dark hair. She denies being intemperate. Her temperature is normal; her pulse is 80 and regular; her respiration is 18, excepting a few bronchial râles at base of the lungs; physical examination is negative. Prescribed: ℞ cascara cordial ℥iiiss, spts. æth. co. ℥ss, liq. strych. br. ℥ss. M. Sig. Tablespoonful, t. i. d.

July 27th. Temperature normal; pulse 84. There were several copious movements of her bowels, the pain markedly less; did not sleep during the night and feels nervous. She complains of a "lump" in her throat and fears sudden death. To take ℞ elix. valerian ammon. ℥ss, syr. brom. co. ℥ij, elix. calisaya c. ferriet strych. ℥iss. M. Sig. Teaspoonful every two or three hours during the day, and bromidia q. s. to produce sleep at night.

July 28th. Temperature normal; pulse 74, full and regular; respiration 18. She slept all night after taking a teaspoonful of bromidia. She feels much better; the pain is gone, and the "lump" don't trouble her much, but she has anorexia. Continue the mixture of the bromides and take a teaspoonful four times daily of ℞ tr. cinchonæ ℥iiss, elix. calisaya co. ℥iss. M.

July 30th. Pulse 74; respiration 18. She has some appetite, and no pain. The "lump" has not returned, and she feels well; her bowels are regular. The report is similar for the two following days.

August 2d. There is a severe burning pain under the left breast, pain in bowels and diarrhœa, profuse sweating. Her temperature is 99.5° F., pulse 96, feeble, and respiration 24. Ordered: ℞ spts. chloroformi ℥ss, tr. capsici ʒi, syr. zingiber. ℥ij. M. Sig. Teaspoonful hourly until relieved, and then a teaspoonful four times daily of ℞ syr. brom. co. ℥i elix. calisaya c. ferri et strych. ℥iij. M.

August 3d. Diarrhœa is checked, and there is no pain. She returned from a visit in the latter part of August and reported having had a good appetite, sleeping well, and having no pain anywhere. She was able to run up or down stairs without fatigue or shortness of breath, and the "lump" came only a few times.

September 1st. At 7 P.M. I was again called to see her. Her temperature was 99.5° F., pulse 84, respiration 22; had "some chills" and a "queer feeling" at the heart, and "an indescribable feeling along the wind-pipe;" there is an irritable cough. Physical examination negative, except a few posterior bronchial râles. Take ℞ quin. sulphate ʒij, morph. sulph. gr. ij, fl. ext. licorice ℥iv. M. Sig. Teaspoonful every two or three hours.

September 6th, 9 P.M. Have not seen patient since last date. She said she felt "good after taking the last medicine, but the cough did not stop." Temperature 98.8° F., pulse 82, respiration 24; physical examination revealed only a few broncial râles at base of lung. There is some dysphagia and anxiety. Placing her hand under the left breast she said, "I can't get my breath when the pain comes here; this will kill me." Firm pressure relieved the pain. Take ℞ fl. ext. grindelia rob. ℥iss, tr. opii camph. ℥ss, cascara cordial ℥iss. M. S. Dessertspoonful every two hours during the night.

September 7th, 10 A. M. Temperature 98°F.; pulse 78, regular; respiration 22 and easy; profuse perspiration; bowels moved twice; the "lump" is troublesome again, and a choking sensation is complained of. The cough is rasping, spasmodic, and at intervals very annoying; but aside from posterior bronchial râles there are no abnormal sounds discoverable anywhere. Consultation was requested, and family declined it.

September 8th, 9:15 A.M. Her husband called at my office, saying his wife had an "awful pain" under the left breast, and had a "regular ague chill;" breathing as usual, no cough, and sweating. Wrote ℞ quin. sulphat. ℨi, pulv. opii gr. x, calomel gr.v. M. Ft. cht. No. x. Sig. One every two hours in water. One hour later I found her sitting in bed vomiting, probably from taking a powder in a *dry* wafer. I gave a powder five minutes later in a *wet* wafer, which was retained, and at 10.50 she was easy. Temperature 97.5°F; pulse 84, regular, but weaker than usual; respiration 22; no cough. She now described the pain as "if some one bored a hot iron into my breast under the heart."

At 2:30 P.M. I was hurriedly called, and found her eyes fixed, pupils slightly, but equally, dilated; she was gasping; her pulse 80 and thready. The surface of her body was very pale and clammy, her extremities icy cold. Whisky hypodermically, friction, etc., failed, the pulse disappeared, a gasp, followed by a long-drawn sigh, a slight tremor, and the curtain dropped on her last act at 2:40 P.M.

I certified *fatty heart* as the cause of death. But owing to subsequent rumors of foul play on the part of the husband, I requested a post mortem examination to settle the question. Sixty hours after death the autopsy was made by Drs. Scribner and Eidemiller, under supervision of the coroner and in presence of myself, Dr. Martin, health-commissioner, Dr. Miller, a representative of the family, a detective and the undertaker.

The body well preserved; adipose tissue under skin nearly an inch thick; no marks.

Thorax opened in the usual way. Right pleural cavity filled with liquid blood, and two blood-clots each the size of a goose egg lay at the base of the right lung near the spinal column. Left pleural cavity contained a very small quantity of serum.

The great vessels of the neck and œsophagus were tied and cut off and a double ligature was placed around the gullet and aorta just above the diaphragm and the tubes severed between the ligatures to facilitate removal *en masse* of the thoracic organs, which was done under difficulties owing to numerous strong old adhesions of the lower lobe of the left lung to the costal pleura and diaphragm. The right lower lobe was also bound down by adhesions but less firmly than the left. Both lungs were exsanguinated, very pale, and the lower lobes presented old inflammatory changes—the left the most.

The pericardium contained a very small quantity of serum —no adhesions.

The heart was imbedded in adipose tissue, small, flabby, and valves normal.

Ascending aorta normal; on the inferior (concave) surface of the middle of the arch was a sausage-shaped swelling, an inch and a half thick, extending downward nearly to the diaphragm, the œsophagus lying in front, the thoracic aorta behind. The gullet was adherent to the tumor and the lower half deeply imbedded in the sac. At the upper right lateral surface of the tumor, just beneath the descending portion of the arch, was an opening into the sac large enough to admit a finger, while the intima was perforated in the middle of the (concave) arch. At the point of rupture the sac wall was very thin and friable; the sac contained fibrin, thrombotic detritus, and a recent clot in its lower portion. The adventitia formed the outer wall of the sac; the media was partly destroyed; there was no further lesion in the intima. Other portions of the aorta were normal; the innominate, carotid and subclavian arteries were free from disease.

Stomach normal, empty; spleen normal; liver normal, gall-bladder full of bile. No other organs examined.

Cause of death: Rupture of dissecting aneurism of the arch of the aorta.

*Remarks.*—Autopsical evidence explains few of the symptoms. Classical symptoms of aneurism—pulsating tumor, bruit or murmurs, eye and voice symptoms, unequal pulse—were absent; dysphagia was a very late symptom, dyspnœa was not marked; palpitation was complained of in July but did not recur. The pain under the left breast was absent more than present and shifted to the right temple; the "lump" and nervousness yielded to tonics and nervines, and did not return for weeks. The dry, harrassing cough was doubtless a reflex act and the globus had a similar origin. The bronchial râles were either caused by reflex irritation or pressure, or both combined. The general picture of the patient during the week preceding death resembled that of malarial intoxication. There were all the signs of neuralgia or hysteria, none of aneurism.

While death was inevitable, vomiting hastened the bursting of the sac at the weakest point by sudden compression and forcible, wavelike, upward propulsion of the sac contents and the finale was—dramatic.

The age of the tumor cannot be accurately given; that it was recent is possible. Theatrical people have a hard, arduous, restless, emotional life with much exposure, and are not free from intemperance.

That the intimate adhesion of the aneurism to the œsophagus caused so little dysphagia is remarkable; and in the absence of a history of illness or injury the lesions of the lungs and pleural adhesions are unexplained.

As the great vessels were not affected or implicated by the perforation in the intima, and as there was no obstruction to the blood current, the regularity of the pulse was not interfered with.

With your permission I now present a condensed description of aneurisms of the aorta within the chest and their treatment. I also present for your inspection the tables arranged for comparison—a brief bibliography is appended, voluminous references are out of place in an essay of this kind.

*Introductory.*—"Aneurism of the aorta develops, particularly in those localities against which the blood-current impinges with special force," says Eichhorst; but dissecting aneurism is an exception to this general rule, and is found only on or in connection with some portion of the aorta, being most common in the thorax. Aortic aneurisms occur in their relative frequency as follows: ascending aorta, arch; descending thoracic aorta, abdominal aorta.*

If a certain area of the aortic wall is weakened a cul-de-sac gradually buds out, enlarges, and the result is a *sacculated aneurism*, on which a second sacculation may develop. If the entire aorta is dilated from any cause for a certain distance a *fusiform* aneurism results, and if a portion of the intima be weakened from any cause resulting in a solution

* Shrady estimates that one death in every 800 is from aneurism. Editorial in the *Medical Record*, July, 1886.

of continuity, through which the blood insinuates itself between the arterial tunics, a sac is formed which may reach to the diaphragm—in one case it reached to the bifurcation of the abdominal aorta. If a local degenerative process of the media causes the intima to yield secondarily the result is a *dissecting aneurism.* If the intima is again perforated at the lower end of the sac and the blood-current re-enters the aortic tube and produces the appearance of a double aorta, the sac wall may be thickened by laminated fibrin within or by adhesive inflammation to adjacent structures without.

The *size* of an aneurismal tumor varies. I have observed numerous aneurisms in the brain smaller than a millet seed. In the larger arteries they vary from the size of a pea to that of an adult head. Baumberger's case of aneurism of the aortic arch projected upward so far that the patient's chin rested on the tumor.

Aneurisms frequently destroy the sternum, ribs or spinal column by erosion, or the inflammatory action resulting from continuous pressure may produce adhesion to neighboring structures, and pressure upon the nerves may cause pain, dyspnœa, etc., and compression of the œsophagus may cause dysphagia.

*Frequency.*—Lebert and Meyer claim that aneurism most frequently involves the arch, but authorities generally give the first place to the ascending aorta, and statistics bear them out. Of Crisp's 551 cases of aneurism, 175, or 31 per centum involved the thoracic aorta, and of 167 of these, 98 involved the ascending aorta, 48 the arch and 21 the descending aorta. Hodgson's collection of 63 cases contains 21, or 33 per centum of thoracic aneurism. In Sibson's collection of 880 cases, 460, or 52.3 per centum involved either the ascending aorta or arch. Of his 703 cases of aneurism of thoracic oarta

87, or 12.37 per centum were within the pericardium; 193, or 27.45 per centum involved the ascending aorta; 140, or 19.91 per centum involved the ascending aorta and arch; 120, or 17.079 per centum, the transverse aorta; 20, or 2.84 per centum, the transverse and descending arch; 72, or 10.24 per centum, the descending arch, and 71, or 10.09 per centum, the aorta below the arch. Hence, 622, or 88.47 per centum of these 703 cases of aneurism affected the ascending aorta and the arch; 420, or 59.7 per centum affected some portion of the ascending aorta, and 280, or 39.82 per centum involved some portion of the arch. Of 109 cases collected by Meyers from the reports of his regiment, and from Netley Hospital, 75, about 70 per centum, affected the ascending aorta or arch. This table will show at a glance the relative frequency of the different portions of the aorta affected.

Aneurisms involving the convex portin of the arch frequently implicate the innominate, carotid or subclavian arteries, but judging from exhaustive researches aneurism of the central portion of the concave (inferior) surface of the arch must be very rare, as I failed to find a single case like mine.

*Age.*—Under the age of 20 years aneurism is not common, and only about 5 to 6 per centum are found under that of 30. The largest number of cases occur between the ages of 30 and 50 years; Lebert, however, says that they are most common between the ages of 45 and 60 years. Garland's 69 cases appear as follows:

| | Per centum. | |
|---|---|---|
| Between the ages of 20 and 30 years, 4 cases | 5.65 | |
| Between the ages of 30 and 40 years, 21 cases | 30.43 | 72.46 |
| Between the ages of 40 and 50 years, 29 cases | 42.03 | |
| Between the ages of 50 and 60 years, 14 cases | 20.29 | |
| Between the ages of 60 and 70 years, 1 case | 1.45 | |

Crisp's cases may be tabulated as follows (omitting indefinite cases):

| | | Per centum. |
|---|---|---|
| Between the ages of 10 and 20 years, | 5 cases | 0.994 |
| Between the ages of 20 and 30 years, | 71 cases | 14.11 |
| Between the ages of 30 and 40 years, | 198 cases | 39.56 |
| Between the ages of 40 and 50 years, | 128 cases | 25.64 |
| Between the ages of 50 and 60 years, | 65 cases | 12.92 |
| Between the ages of 60 and 70 years, | 25 cases | 4.97 |
| Between the ages of 70 and 80 years, | 8 cases | 1.59 |
| Between the ages of 80 and 90 years, | 2 cases | 0.397 |

Sacculated or fusiform aneurism is more common in middle age, while dissecting aneurism is said to increase with advancing age (Peacock). My case was 32 years of age. Broca claimed that the liability to aneurism of the aorta above the diaphragm increased with advancing years, while the opposite obtained below the diaphragm.

*Sex.*—External aneurisms are most common in males, and internal aneurisms of the circumscribed form are most frequent in males, (?) being from 66.66 to 80 per centum (Blakiston, Crisp, Peacock). In 82 cases Garland found one seventh, Crisp found less than one eighth, and Hodgson about one ninth in females. In the Registrar-General's report (Great Britain, 1874) 685 deaths are attributed to aneurism, of which 537, or 89.5 per centum were males. Of 21 cases of dissecting aneurism 14 were females and 7 males.

Holmes believes that internal aneurisms are common among women whose way of life exposes them to the vascular excitement following intemperance, vice or mental emotions; and Rendle's statistics of internal aneurisms among female convicts show conclusively that mental depression and excitement are potent predisposing factors.

*Aetiology.*—The causes of aneurism may be mechanical, local or constitutional. A fall, blow, push, concussion of the body, running, jumping, heavy lifting, strains, sudden interference with respiration, or stretching or compression of the neck after violent exertions, are potent factors if the aortic tunics are weakened from any cause. Injury of the wall of the vessel by stab, shot or other wound may be followed by traumatic aneurism. Certain employments requiring powerful exertions or violent straining causing over-distension of the aorta leading to slow atheromatous inflammation (Moxon) may cause aneurism. Tight lacing, constriction of the neck by tight collars or accoutrements (Frotnute). In the British army the deaths from aneurism exceed eleven times the mortality from the same disease in civil life, and this enormous death rate was attributed to the old-fashioned stock or collar (Meyers). Exposure, intemperance, severe or long continued mental emotions or excitement are Aetiological factors; continued intra-vascular pressure tends to weaken the walls of the vessel.

Mechanical impediments beyond the arch, loss of muscular tone in the tissues, embolus, vaso-motor palsey (Rokitansky) is denied by Niemeyer. The result of a ligature of a vessel tends to dilate the wall of the vessel.

Hereditary predisposition is questionable, but Fuller relates the history of a case whose paternal grandfather, uncle and father had all died of aneurism, and whose sister was afflicted with that disease.

The aneurismal diathesis is an improbable factor, but a case in which sixty-three aneurisms were found is on record.

Fatty degeneration, atheroma, calcific spots whose plates have sharp edges which wound the intima, chronic endarteritis, are direct causal factors. Ulceration or abscess of the wall of the vessel endarteritis even primary inflammation or atrophy of the external or middle coat of the artery may cause aneurism. The most prevalent seat of chronic endarteritis is in the ascending aorta and arch, and rupture or aneurism is most common here.

Aortic regurgitant disease following rheumatic endocarditis may cause aneurism. Powell saw a lad aged 17 years suffering from aortic aneurism which could be traced only to two rheumatic attacks in his eleventh and twelfth years.

Gout and syphilis dispose to degenerative processes in the tunics of the arterial coats. George Ross asserts that fifty per centum of the aneurismal cases have had syphilis. It is remarkable that aneurism is thirteen and a half times more prevalent per 1,000 men in the British army than in the navy (Barwell). Certainly no one will maintain that sailors are more virtuous than soldiers. Both are subject to great hardships, violent exertions and exposure at certain times; but the soldier's uniform stretches and constricts the neck, keeping him in an unnatural and strained position, while the sailor's clothing is loose and his training gradual.

Any constitutional trouble which exerts a degenerative influence on the arterial coats may be a factor in the causation of aneurism. *Symptoms* are usually absent in the beginning of this disease, and death may result without the least suspicion of aneurism. Free living, intemperance or violent exertions may be followed gradually by palpitation, dyspnœa, deep-seated pain in the chest over which there may be a pulsating tumor and a bruit. The tumor may compress the air-tubes, œsophagus, neighboring blood vessels or the vagus and recurrent laryngeal nerves, and dropsy, discoloration, pain, altered voice, throat symptoms, disordered vision, headache, blood-spitting, may be produced, erosions of the bony walls of the chest may follow and palsy or paraplegia may obtain before death results.

The *pain* may be dull, intermittent, with exacerbations, or persistent, hot, boring or burning in character, frequently agonizing and preventing rest or feeding; it may be seated over the tumor, under the left breast, at distant points and may shift from one point to another.

*Dyspnœa* is present when the aneurism compresses the air-tubes, or when innervation of the larynx is interfered with. The intrinsic muscles of the larynx are expiratory (Sanderson) and section of the recurrent laryngeal nerves paralyses the vocal cords which are sucked together at each inspiration (Reid, Ligallois). Compression of one of the pulmonary plexuses may produce dyspnœa resembling asthma (Gairdner). Irritation of the pneumogastric or any of its branches may cause spasmodic dyspnœa. The tumor may narrow the trachea or bronchi producing an accumulation of mucus at the narrowed point, hence an obstruction, and difficult breathing follows (Bristowe).

*Dysphagia* is not a prominent symptom and may be absent, although the œsophagus is compressed by the aneurism (Leudet); the gullet may be opened by erosion from the tumor and difficult swallowing may not be present (Fuller, Quain). In my case dysphagia was a very late symptom.

*Palpitation* and irregular or uneven pulse will be noted when the tumor or sac interferes with the normal flow of the blood current through the aorta or vessels which spring from the arch. Nervous reflex irritation may cause palpitation. In my case the pulse was never irregular or uneven nor more than ninety-six at any time, but cases are reported with a pulse from 130 to 140 beats per minute (Powell).

*Headache*, if present, may be severe, paroxysmal, continuous, and may simulate neuralgia, migraine or clavus.

*Hæmoptysis* is rare during the progress of aortic aneurism, but may become a marked symptom before death. Gairdner mentions a case in which bloody expectoration preceded death for some time, and Blakiston records a case in which fragments of discolored coagula were expectorated with blood several weeks before death. (Diseases of the Heart.)

*Unequal* pupils must be received with caution, as one person in every fourteen has one pupil smaller than the other. (Robertson.) If the myosic and immovable pupil be present it is on the same side of the body as the aneurism; it is not a prominent symptom. Powell observed it in four cases out of thirty-six in aortic-arch aneurism; and Walshe rarely observed pupillary changes either larger or smaller in this disease.

By *inspection*, large, tortuous veins on the side of the neck and humeral region will be observed if any of the larger veins are compressed; the face may be reddened, there may be œdema or dropsy of the upper or lower part of the body; pulsation in the intercostal spaces if the aneurism closely approach the chest wall; the antero-posterior diameter of the upper part of the thorax may be increased, the superior portion of the sternum prominent, rounded and mottled with blue veins. There may be a cyanotic color in the face, neck or chest. If the tumor is very large the sternum and ribs may be displaced outwards, and in extreme cases the chest wall may he destroyed by erosion and the tumor projects externally.

*Palpation* will detect changes in the coats of the blood vessels and a difference in pulse-beat on the two sides of the body (if present). It may reveal two centers of synchronous pulsation within the chest with a perceptible thrill over the aneurism, which heaves and expands in every direction, and if distinct from the heart is diagnostic; but such an aneurism must be near the surface. If the interior of the sac-wall is thickened by laminated fibrin the thrill may be lost and the aneurism then resembles a tumor lying on the aorta. In obscure cases "pressure with the flat of one hand on the anterior part of the chest while the other is placed between the shoulders during expiration" (Stokes) will aid in detecting deeply-seated impulse expanding in every direction. A distinct thrill felt with the beat of the tumor is not common. Powell observed it only eight times; Hope never saw a case of aneurism with thrill, and Walshe considered thrill a common sign in peripheral dilatation.

*Percussion* will reveal dullness or increased resistance if the aneurism is near the surface, of large dimensions, lined with laminated fibrin or coagula, or solidified. When the tumor is deeply seated or small, percussion will be either indefinite or negative.

*Auscultation* may reveal only a few bronchial râles, as in my case, or a systolic murmur accompanied by a bruit may be heard over the tumor. All auscultatory signs are most distinctly heard over the aneurism and may be conducted along the aorta and heard in the interscapular region. The bruit is not a reliable sign. Long ago Parkes pointed out the importance of ascertaining whether the bruit replaces one of the heart-sounds (Clinical Lectures). In about one-third of the cases of aneurism there is no audible bruit; Powell noted its presence in twenty-two cases of thirty-six reported, it being double in twelve cases. Lebert claims to have observed it in the earlier stages of one-half of his cases. In dissecting aneurism this sign is absent.

*Diastolic shock*, when present, is significant of aneurism and an absolute sign if it follow a systolic impulse in disease of the ascending or transverse aorta. Powell noted this sign fourteen times in thirty-six cases, and it was also twice observed in aneurism of the abdominal aorta. (Balfour, Wickham, Legg).

*Diagnosis.* The sign-center for aneurism of the ascending aorta is in the second right intercostal space close to the sterum. Cyanosis and dropsy of the upper part of the body supervene if the innominate vein or ascending vena cava is compressed. As the tumor enlarges the heart is displaced downward and the area of pulsation enlarges downward and to the right (Gibson).

The sign-center for the arch is the manubrium sterni towards its left edge. A forward projection of the tumor is not common; backward the tumor may press upon the trachea, bronchi, œsophagus, venæ cavæ, vagus and recurrent nerves. If the tumor springs from the concave surface of the arch and projects downward, there may be vague and indefinable signs, peculiar sensation along the trachea, globus, irritable cough, slight dyspnœa, and dysphagia when the tumor has somewhat enlarged. Pain at the apex of the heart may simulate cardiac neuralgia, intercostal neuralgia, pleurodynia, hysteria, etc. In dissecting aneurism involving the concave (inferior) surface of the arch unequal pulse is absent.

The sign-center for the descending portion of the arch is the left upper interscapular region. There may be severe pain in the back and shoulders, pulmonary dyspnœa, spasmodic in character, or diminished respiration and dullness at the base of the left lung. Very large aneurisms may cause erosion of the vertebræ and pressure upon the cord. There may be dysphagia, throat and eye symptoms.

Aneurism of the thoracic aorta below the arch is not very common, and is difficult to discover if small. Laryngeal symptoms are not present. Severe and continuous pain may be the only symptom. If the tumor is large, curvature of the spinal column and anterior displacement of the heart will be noted and the œsophagus, bronchus, or portions of the left lung will be pressed upon. There may be pulsation and dullness over the tumor, and pressure upon the ascending cava will produce enlargement and tortuosity of the superficial veins, and œdema or dropsy of the lower extremities. Erosion of the spinal column may supervene and may produce most agonizing pain, palsy or paraplegia.

Aneurism of the convex portion of the arch frequently implicates the innominate, carotid or subclavian artery. It is frequently impossible to differentiate between aneurism of the arch simple, and aneurism implicating one of the vessels springing from the arch. Aneurism of the arch may be mistaken for innominate aneurism and *vice versa.* There are no absolute signs to differentiate one from the other. In a collection of twenty-four diagnosed innominate aneurisms (Barwell) more than thirty-three per centum proved either aortic aneurism or aortic dilatation on autopsy.

In dysphagia resulting from an obscure cause the œsophageal sound should be sparingly and cautiously used to avoid penetration and rapid death from hæmorrhage.

Sphygmographic tracings will aid in the diagnosis in many cases, by showing the decreased blood pressure on one side.

*Differential Diagnosis.*—"Aortic aneurisms may be one of the easiest problems in clinical medicine," or may present "difficulties which defeat the most skillful diagnostician." "It may be diagnosed as present" and *vice versa* (Garland). Many aneurisms show no signs of their presence

(Guttmann), and their murmurs frequently disappear temporarily or permanently (Eichhorst).

There is only one positively characteristic phenomenon of thoracic aneurism and that is the existence of a tumor in some part of the chest which beats isochronously with the heart, and which expands in every direction (Balfour). The diagnosis can rarely be made with certainty (Powell), for so long as an aortic aneurism has not obtained considerable dimensions, and has not touched the chest wall, characteristic symptoms are absent (Guttmann).

An aneurism is a tumor and many of its symptoms are signs of a tumor. It may pulsate from lying on or over the aorta, therefore it is essential to determine whether the tumor is solid or is an expanded vessel—a cystic tumor or large abscess lying on or over the aorta will present symptoms of aneurism and the diagnosis will be uncertain. A solid tumor may pulsate, but does not expand (Balfour), there is no bruit of a booming character (Hayden), nor accentuation of the second sound of the heart (Walshe). Aneurismal tumors change their size and mural tension (Peacock;) other tumors are not so affected. The following points will aid in diagnosis:

1st. Aneurismal tumors may rise and fall during coughing, swallowing or straining, and simultaneously expand in all directions.

2d. Under 25 years of age and absence of injury is against aneurism.

3d. Great emaciation with absence of internal pain negatives aneurism.

4th. Great cardiac displacement and absence of a large pulsating tumor negatives aneurism.

5th. Severe continuous or paroxysmal pain in the chest favors aneurism.

6th. Unequal pupils, irregular pulse in the radials or carotids, dysphagia, laryngeal or bronchial dyspnœa favor aneurism.

7th. "Absence of signs indicating ordinary heart or lung affections in an individual suffering persistent anomalous disturbances within the thorax, although he does not exhibit any failure of general health, affords strong suspicion of aneurism" (Walshe).

That diagnosis is frequently extremely difficult or impossible is shown by a host of cases, from Riles (10), Crisp (7), Smith, Bell, Guthrie, Feame, Pelletan, Dupuytren, Barwell and others, cases taken for troubles other than aortic aneurisms, and *vice versa.* In 1865 Heath tied both the right carotid and subclavian arteries for supposed innominate aneurism, and the autopsy four years later proved it aortic aneurism. Fenwick's case was diagnosed innominate aneurism and section proved it wholly aortic. My friend Prof. Senn relates a case diagnosed thoracic aneurism which on post-mortem examination proved to be a solid tumor overlying the aorta. Mahomed and Golding Bird report a case of abscess of a gland in the episternal notch taken for aneurism. In a case of innominate aneurism the symptoms were so vague that the diagnosis was made only when the sac was laid bare by incision. In another instance the presence of a tumor in the episternal notch, with double murmurs, pulsation, and pressure symptoms, on section proved to be a vascular tumor of the *manubrium sterni.* One of Guttmann's cases presented all the classic symptoms of aortic regurgitation and none of aneurism, in which section showed the aortic valves intact, and revealed a large aneurism of the ascending aorta. Dujardin-Beaumetz's case presented contraction of the left pupil, sudden reddening of the left half of the face, transient aphonia, intermittent dyspnœa, suppression of left radial pulse, and a double souffle along the aorta, and the necropsy showed simple endarteritis of the arch without dilatation.

Inflammatory conditions implicating the vagus nerve or its thoracic branches may produce symptoms of the eye, throat and vascular system resembling aneurism.

Chronic empyema of the pleural cavity may simulate aneurism, but absence of bruit, more extensive dullness, irritative fever and pointing of the abscess would be distinctive signs. Birard saw a case of this kind in which a tumor to the left of the sternum, which appeared and pulsated like an aneurism, subsequently discharged pus.

Retraction of the margins of lung tissue overlapping the heart and aorta—presenting dullness, forcible local pulsation, bruit, palpitation and dyspnœa—has been mistaken for aortic aneurism (Powell), and aortic regurgitant disease may simulate aneurism (Hope).

*Prognonis.*—In the large majority of cases aortic aneurism is fatal, and "the uncertainty of the time and suddenness of death give this disease a peculiar terror," and frequently a dramatic ending. For while "the sac-wall may be thickening by fibrinous layers at one point fatal erosion may proceed at another."

*Duration.*—The progress of anuerism is rapid and in most cases fatal within a few years. From a large number of observations Lebert places the average duration from six months to four years. In 40 cases of which the duration of the disease was well known 20, or 50 per centum, died during the first year, 9 lived 2 years and 3 lingered 5 years (Garland).

Peacock and others limit the time to five years; but many cases are not diagnosed during life, others only shortly before death, and hence an exact limit cannot be given.

*Mode of death.*—In many cases the direct cause of death is rupture of the aneurism into cavities, vessels or tubes. In a series of 106 cases 39 terminated by rupture, and 13 of these or 33 per centum burst into the pericardium. Of Thuman's 18 cases of thoracic anuerism 11 communicated with the pulmonary artery, 4 with the cavity of the heart, and 3 with the venæ cavæ. Of 42 cases collected by Sibson and Barwell:

Seventeen cases of aneurism of the ascending aorta communicated with the pulmonary artery.

Six cases of aneurism of the ascending aorta communicated with the right auricle.

Three cases of aneurism of the ascending aorta communicated with the right ventricle.

Four cases of aneurism of the ascending aorta communicated with the descending cava.

Three cases of aneurism of the ascending aorta communicated with the left ventricle.

Two cases of aneurism of the transverse aorta communicated with the descending cava.

Seven cases of aneurism of the descending aorta communicated with the ascending cava.

Ascending aortic aneurism, may burst into the right pleural cavity or pericardium externally. Aneurism of the arch may burst into either pleural cavity, the trachea, the bronchi the œsophagus, the lungs, the mediastina, or externally. Aneurism below the arch may burst externally into the left pleural cavity, the œsophagus, or the spinal canal.

Rupture of an aneurism is not always immediately fatal. The so-called "weeping aneurism" may pour out small quantities of blood for weeks. Neligan's case discharged blood through an opening near the second rib on the right side for more than a year, and the hæmorrhage was finally checked and the tumor solidified. In another instance a patient mistook an aneurism for a "bloodboil," and squeezed it with his chin to favor bleeding until he fainted. Hæmorrhage then ceased and did not recur. Aneurism may cause death indirectly by causing secondary emboli or infarcts; by asphyxia, starvation or exhaustion, and from the size or pressure of the tumor. Sudden formation of a soft clot within the sac may result in inflammation and suppuration which may kill the patient before the tumor bursts. A very small number of cases die of acute intercurrent disease.

The more latent the aneurism the more frequently death occurs by rupture.

Notwithstanding Flint's dictum, "that clinical observation shows that patients afflicted with thoracic aneurism rarely have pulmonary tuberculosis," it is evident that interference with respiration has a deleterious effect on the lungs, for in Harrot's collection of 77 cases phthisis was established as present in 18 cases of 42 examined. The remaining 35 cases were not examined to discover lung changes.

*Treatment*, in many cases of thoracic aneurism, is utterly futile, but in a large number euthanasia is desirable to relieve the pain and to improve the general health. The first step in treatment is absolute rest in the recumbent or semi-recumbent position. Worry, excitement, anger and violent mental agitation may prove rapidly fatal by syncope or rupture of the sac, and hence there must be mental quietude. Stimulants must be prohibited, the diet carefully regulated, the bowels kept soluble and the kidneys active. A long rest has more than once shown remarkable results. It is difficult to carry out surgical measures, and the treatment promising best results is rest and such remedies as may be required. The ancient treatment was copious venesection, frequently carried to syncope, which was supposed to possess some occult power to cause coagulation within the sac (Thackrah, Gulliver and others).

Valsalva (1748) treated aneurism by phlebotomy and enough diet to starve his patients, and forty days rest in bed was rigorously carried out. Tufenell improved Valsalva's method, by omitting venesection and giving either low diet or dry diet. The results from this treatment are encouraging, and it deserves repetition with appropriate remedies added.

The number of drugs or remedies employed and recommended for aneurism is legion, but most of them are utterly useless. It is well to remember that thoracic aneurism is fatal within a certain time, and that the patient's distress must be relieved; to prolong his life and relieve his suffering being our aim. The remedies may be arterial sedatives, nervines, anodynes, alteratives, and probably tonics in some cases.

Intra-vascular pressure must be reduced, and the pulse brought down to 60 and kept there. The best remedy is tincture of aconite root in small doses frequently repeated. Aconite not only reduces pulse and blood pressure, but benumbs the nervous system as well. Toxic doses may not do so well as in Prof. Pancoast's case, in which a subclavian aneurism was cured by an overdose of aconite taken by accident! Tincture of veratrum viride, or veratria ointment, may be used, but both are powerful depressants, and vomiting and collapse must be avoided. Gelsemium is a good sedative and relieves pain. Digitalis is a dangerous drug in thoracic aneurism, and should not be used, because of its cumulative tendency aud of the increase of the heart's force which it causes. Belladonna, or atropia, is useful in small doses, and is highly extolled in Europe. To control nervous reflex irritation the bromides and dilute hydrocyanic acid stand at the head of the list. Chloral hydrate or urethan may be used to overcome insomnia, anodynes to assuage pain, but opium or its salts should not be too freely exhibited. Large doses of the bromides are required to control the nervous system at times, and they should be exhibited until the system is well under control, when the dose may be reduced or given at longer intervals. The writer has frequently taken four drams of the bromide of potassium at a single dose to relieve an agonizing migraine, from which the relief was prompt, within an hour or two, and no bad after effects followed.

Ergotin was successfully employed by Langeubeck (1869) by parenchymatous injections near the aneurism. It reduced the size of the tumor, lessened the pulsation, and markedly relieved the pain. Dutoit (1871) and Carlin (1878) had equally favorable results. The fluid extract may be given internally.

Tannin and acetate of lead have been recommended, but aside from astringency their therapeutic power over aneurism must be small indeed.

Iodide of potassium was first prescribed in cases of aneurism by Chutterbutty, of Calcutta (1862), and recommended by Roberts (1863) and championed by Balfour a little later. At the present time it is generally considered a sort of specific in internal aneurism. Berènyi cured (?) his case of aortic aneurism in two months by ordinary doses! Cayley's case was not benefited by "diet and iodide of potash." Whether the iodide causes increased coagulation of the blood, or produces a slow thickening of the sac-wall (Bramwell) is not material. Its alterative and "dissolving effect" on syphilitic tissue changes have made this salt a so-called specific, and since aneurism is frequently the sequence of venereal infection the iodide may be valuable on that score. But judging from the results of a host of cases treated by this drug it is evident that rest, diet and total abstention from stimulants received no consideration in the results obtained; and, further, when the patient threw off restrictions and indulged in stimulants, plentiful feeding, or exercise, the disease advanced rapidly in spite of large doses of the iodide. It is very difficult to prove its "specific value" in aneurism, for the potassic iodide cannot be given alone while everything else is withheld. Sir William Gull and Holmes consider it of little value, and Barwell says, "I have not found a single case of aneurism cured by iodide of potash." It is a useful remedy in this disease, but overrated, unless, we repeat, aneurism is caused by or results from syphilitic infection, and if so mercuric preparations singly or combined with potassium iodide will be doubly valuable.

Surgical treatment of thoracic aneurism has, with few exceptions, proved fatal in a very short time. Double distal ligature of the right carotid and subclavian was successfully performed by Heath, and patient survived four years; by Barwell, whose patient survived fifteen months; by Wyeth, whose patient died a year later of diarrhœa; by Lediard, whose patient died about ten months later; by Kelly, whose patient was alive at last accounts, and unsuccessfully by Ashhurst, who tied the right common carotid, and death occurred next day.

Pressure or compression are, of course, out of the question, and cauteries as employed for aneurism of the anterior palatine artery (Chassaignac), and of the palm (Newmann), are not applicable in aortic aneurism.

Monteggio's method of injecting coagulating liquids should not be attempted, as the danger from embolism, infarcts, etc., is too great.

Dieulafoy and Marsacci injected a weak salt solution, 1:20, into the cavity of the aneurism, but the results were far from brilliant, and the method is useless. Acupuncture originated with Home (1796), who expected that a foreign body within the sac would cause a coagulum, but it led to no practical results (Velpeau, Pravaz, Agnew, and others). MacEwen was more successful. Heath introduced three pairs of needles, interlacing each other, into a subclavian aneurism and left them four days, the result was death; tumor solid (?). Mialago's cases all ended fatally shortly after the operation (1850–5). Rizzoli's first case of aneurism (at the elbow) on withdrawal of needles required amputation to check hemorrhage. In his second case (ilio-femoral) digital compression failing, acupuncture reduced the size of the tumor and lessened the pulsation. Murray's second case (aortic aneurism) was twice punctured, and erysipelas developed in the punctures and caused death. His first case (aneurism of the arch and innominate artery) was treated with (18–20) needles left *in situ* twenty-four hours, and then twenty-four feet of fine iron were introduced. Sudden death occurred two weeks later. His fourth case (saccular subclavian aneurism) was completely transfixed with (20–30) long needles at intervals, causing consolidation, decrease in size of tumor, and relief from pain.

Galvanism was added to acupuncture by Benjamin Phillips (1829) in England and established in France by Pravaz, and perfected by Ciniselli, Duncan, Fraser, Poore, Althaus et al. In fusiform aneurism galvano-puncture is useless, but it may be beneficial in sacculated aneurism. Cinicelli treated thirty-seven cases of aortic aneurisms by galvano-puncture and cured six. Poore's eight cases of thoracic aneurism ended fatally by same treatment. Dujardin-Beaumetz's case of thoracic aneurism died from electrolysis. Black's case of thoracic aneurism died from galvano-puncture, and at the autopsy no coagula were found. Barwell punctured the sac-wall of a thoracic aneurism with a hollow ivory needle, through which he introduced ten feet of steel wire which he connected with the positive pole of a galvanic battery, using a current of about nine or ten milliamperes, and death occurred a week later, with little coagulation.

Whether the needles or the galvanic current produce the best effect it is difficult to determine, but a foreign body produces the same effect in the aneurism whether attached to a battery or not (Heath, Marshal, MacEwen), and the whole benefit derived from galvano-puncture is caused by inflammation and thickening of the sac-wall (Paul). But the dangers of galvano-puncture (Barwell), hæmorrhage, eschars, inflammation of the sac-wall, possible suppuration, embolism, infarcts, etc, are so formidable and the result so uniformly fatal that the risk is too great.

Fine iron wire, twenty-six yards, were introduced through a fine canula into an aneurism of the aortic arch, which had destroyed a portion of the bony wall of the chest, by Moore, in 1864. The operation was easy and almost painless. Inflammation of the sac and surrounding structure, emboli, infarcts, and great pain followed, and death occurred on the fifth day, and the wire was found to be imbedded in a clot.

Bacelli introduced fine watch-springs into the cavity of the aneurism, and both of his cases proved fatal in a short time.

Murray (third case) introduced twenty feet of catgut into an aneurism of the arch and innominate artery. Death occurred, the catgut was softened and no coagula was found.

Horse-hair (twenty-four feet, nine inches) was introduced into a right subclavian aneurism by Levis (1873). Death occurred from rupture of sac and the hair was found imbedded in a clot.

Bryant introduced thirty feet of horse-hair into a popliteal aneurism and death on fourth day; the tumor being almost solid.

Stimson introduced fifteen pieces of horse hair, each six inches long, into an ilio-femoral aneurism without affect on the tumor.

From the foregoing it is evideut that the introduction of foreign bodies into the cavity of an aneurism is generally followed by disastrous results. The practice is pernicious and almost invariably fatal within a very short time. A clot may be found about the coils, but the complications are many, varied and fatal. Thoracic aneurisms are best managed by perfect rest, carefully regulated diet, total abstinence from all stimulants and the use of such remedies as may be necessary to control the circulation, assuage pain, produce the requisite amount of sleep, and if possible, to impress the disease and check its march to a fatal issue.

BIBLIOGRAPHY.

Ashhurst, Surgery; Chelius, Chirurgie; DaCosta, Medical diagnosis; Eichhorst, Krankheiten d. Resp. u. Circ. Org.; Flint's Practice of Medicine; Flints, Clinical Medicine; Hall, Medical Diagnosis; Holmes, System of Surgery; International, System of Surgery; Niemeyer, Practice of Medicine; Pepper, System of Medicine; Quain, Dictionary of Medicine; Reynolds, System of Medicine; Real, Encyclopæd. d. Gesamt. Heilkunde; Reference, Hand-book of Medical Sciences; Wagner, General Pathology, and from Personal Communication; Medical Journals and other sources.

296 West Water Street, Milwaukee.

